# Primary care transformation in Scotland: qualitative evaluation of the views of national senior stakeholders and cluster quality leads

**DOI:** 10.3399/BJGP.2022.0186

**Published:** 2022-09-21

**Authors:** Eddie Donaghy, Huayi Huang, David Henderson, Harry HX Wang, Bruce Guthrie, Andrew Thompson, Stewart W Mercer

**Affiliations:** Usher Institute, College of Medicine and Veterinary Medicine, University of Edinburgh, Edinburgh, UK.; Usher Institute, College of Medicine and Veterinary Medicine, University of Edinburgh, Edinburgh, UK.; Usher Institute, College of Medicine and Veterinary Medicine, University of Edinburgh, Edinburgh, UK.; School of Public Health, Sun Yat-Sen University, Guangzhou, China.; Usher Institute, College of Medicine and Veterinary Medicine, University of Edinburgh, Edinburgh, UK.; School of Social and Political Science, University of Edinburgh, Edinburgh, UK.; Usher Institute, College of Medicine and Veterinary Medicine, University of Edinburgh, Edinburgh, UK.

**Keywords:** clusters, general practice, healthcare inequalities, multidisciplinary working, multimorbidity, primary care, primary care reform

## Abstract

**Background:**

Primary care transformation in Scotland aims to improve population health, reduce health inequalities, and reduce GP workload. Two key strategies (formalised in April 2018 in the new Scottish GP contract [Scottish General Medical Services contract], although started in early 2016) are the expansion of the multidisciplinary team (MDT) and GP cluster working.

**Aim:**

To explore progress in the implementation of the GP contract in Scotland in terms of the MDT and cluster working.

**Design and setting:**

Qualitative study with key national primary care stakeholders (PCSs) (*n* = 6) and cluster quality leads (CQLs) in clusters serving urban high deprivation areas (*n* = 4), urban mixed areas (*n* = 4), and remote and rural areas (*n* = 4).

**Method:**

Semi-structured interviews with thematic analysis.

**Results:**

There was general support for the initial aims of the new GP contract but all interviewees felt that progress on both MDT expansion and cluster working was slow, even before the pandemic. None of the CQLs (and few PCSs) felt that GP workload had reduced significantly, nor that the care of patients with complex needs had improved. Lack of time and poorly developed relationships were key barriers, as was a lack of relevant primary care data, and additional support (including guidance, administration, training, and protected time).

**Conclusion:**

Key PCSs and CQLs in different areas of Scotland report limited progress in primary care transformation, only partly related to the pandemic. There is a need for better workforce planning and support if the new GP contract is to succeed in transforming primary care in Scotland.

## INTRODUCTION

As in the rest of the UK, Scotland faces major health and social care challenges including ageing populations, increasing rates of chronic illness and multimorbidity, growing socioeconomic and health inequalities, and other emerging risk factors that have an impact on population health.^1^^,^^2^ Health and social care policy is a devolved responsibility in Scotland, and there have been significant recent reforms that aim to transform primary care and integration of care. The Scottish Government introduced legislation in 2014 to integrate health and social care services, which led to the formation of integrated joint boards (IJBs) in each local authority area and their associated delivery arm, the Health and Social Care Partnerships (HSCPs), in 2016.^3^ Alongside these organisational changes, a new Scotland-only GP contract (the Scottish General Medical Services contract) was formalised in April 2018 after lengthy negotiations spanning several years following the abolition in April 2016 of the Quality and Outcomes Framework (QOF) pay-for-performance programme.^4^

The two key components of the new Scottish GP contract are the expansion of the multidisciplinary team (MDT) and the formation of GP clusters (both of which started in early 2016).^4^ GP clusters are geographical groups of five to eight GP practices who work together, with protected time, to improve the quality of care of their local population (their intrinsic role) and contribute and provide local leadership to the planning and development of integrated care within the IJB and HSCP (their extrinsic role).^5^ Each of the 147 clusters in Scotland has a GP cluster quality lead (CQL) and every practice has a practice quality lead.^5^ The specified aim of the Scottish Government was that CQL roles (which commenced in April 2016) would be functional across Scotland by April 2017.^6^

A key aim of the new General Medical Services contract and wider primary care reforms in Scotland is to reduce GP workload, with the wider MDT staff members taking over clinical work previously undertaken by GPs.^4^ The MDT staff include urgent care practitioners (paramedics), advanced nurse practitioners, advanced physiotherapy practitioners, pharmacists, mental health nurses, and community links practitioners.^4^ The expansion of the MDT is expected to enable GPs to focus on their role as expert medical generalists, dealing with undifferentiated illness, and providing longer consultations to patients with complex care needs, such as those with multimorbidity.^4^

In terms of investment in the new contract, the Scottish Government (in 2018) committed to spend an additional £250 million in direct support of general practice by 2021–2022.^7^ This forms part of an overall commitment to invest an additional £500 million in primary care by 2021/2022.^7^ In 2020/2021 the sum of payments made to the 928 general practices was £950.5 million, an increase of £55.8 million (6.2%) when compared with 2019/2020.^8^ The MDT budget is part of the direct costs and in 2021/2022 was £155 million. Between March 2018 and March 2021, the Scottish Government reported that there were 2463 whole-time equivalent new MDT staff appointed in primary care, with the largest group being pharmacists and pharmacy technicians (>1000).^9^ In December 2017, the Scottish Government also pledged to increase GP numbers by 800 within a decade.^10^ Between 2018 and 2021, GP numbers increased by 209, but this was entirely the result of an increase in female GPs, who mostly work part-time, and the whole-time equivalent number is unknown.^11^

**Table:** How this fits in

Expansion of the multidisciplinary team (MDT) and cluster working are at the heart of the new Scottish GP contract (formally introduced in April 2018) aiming to reduce GP workload and improve quality of care (including for patients with complex problems). However, in 18 qualitative interviews, key stakeholders and cluster quality leads reported slow progress, little or no reduction in GP workload, nor improvements in patient care. Key barriers identified were lack of time, poorly developed relationships, and inadequate support. There is a need for better workforce planning, better primary care data, and more support, if the new Scottish GP contract is to succeed.

To the authors’ knowledge, there has been no published evaluation of the expansion of the MDT in Scotland. A national survey of GP clusters in Scotland conducted in 2018 by the Scottish School of Primary Care found that, although clusters were ‘up and running’, there was a perceived general lack of structural support, training, and capacity building,^12^ which was supported by subsequent qualitative interviews with a range of different stakeholders.^13^ The aim of the present study was to investigate the views of key national primary care stakeholders (PCSs) and CQLs working in three different health board areas of Scotland (high deprivation; mixed/affluent; and remote and rural) about the GP contract and wider primary care reforms.

## METHOD

The present study reports this research using the Standards for Reporting Qualitative Research framework.^14^ The study was conducted and reported in accordance with the consolidated criteria for reporting qualitative research (COREQ).

### Study design

Qualitative methods were used to explore the views of key Scottish PCSs and CQLs in Scotland. Data were collected using in-depth semi-structured interviews. As a result of COVID-19 and lockdown measures all interviews were conducted by telephone, which was commonly done by researchers during the pandemic.^15^

### Sampling and recruitment

This study is the first phase of an ongoing programme of funded research on primary care transformation in Scotland led by the corresponding author. As part of this wider programme, 12 clusters have been recruited across three health boards (out of a total of 14) for qualitative and quantitative evaluation. This number was determined by the funding limit of the research programme. The health boards were chosen to give a range of population characteristics including urban areas of high deprivation (health board one), urban mixed, including affluent and deprived (health board two), and remote and rural populations (health board three). CQLs were approached through the primary care clinical leads in each health board and given details of the programme of research, which they discussed with the practices in their cluster. The research team were available to answer any queries. Recruitment stopped once four clusters were recruited in each health board.

The PCSs (*n* = 6) were senior staff selected from six key national organisations involved in supporting the new GP contract in Scotland. Three of the six were also practising GPs. The health boards and the PCSs’ organisations are not named to preserve the confidentiality of the participants.

### Data collection

One-to-one telephone interviews with PCSs and CQLs, lasting approximately 60–70 min, were conducted by the first two authors between March and May 2021 for PCSs and June and December 2021 for CQLs. Interviews were recorded and transcribed verbatim. Separate, although broadly similar, interview topic guides were developed for the PCSs and the CQLs. For the PCSs, areas covered included their views on the original intention of the primary care reforms and expected outcomes at that time, and their views on progress nationally (particularly MDT expansion and cluster working) including the impact and key learning. The same questions were asked to CQLs but interviewees were asked to locate their replies in the context of their own cluster areas. Additional questions to CQLs were asked on support and training for clusters and CQLs.

### Data analysis

Analysis was thematic^16^^,^^17^ to identify commonalities and differences regarding the relevance and implementation of the contract/reforms in the context of the three diverse population groups in each health board. Three authors (the first two authors and the corresponding author) independently developed initial codes based on individually analysing PCS and CQL transcripts, and agreed on the coding frame through discussion. The transcripts were coded using NVivo (version 12 Pro) by the first author. The phases of thematic analysis outlined by Braun and Clarke^16^^,^^17^ were applied by the core team of researchers in the six following steps: familiarisation with the data; generation of initial codes; searching for themes; reviewing themes; defining and naming themes; and producing the final report.

The agreed themes were also discussed with the wider research team, including the four members of the patient and public involvement group established for this programme of research, and sent to participants for comments.

## RESULTS

Table 1 shows the characteristics of the CQLs who took part in the study from the three different health boards. There was a range of years of experiences as a GP, in general and in their current practice, and as a CQL. The characteristics of the PCSs for reasons of confidentiality are not included, but they were all very experienced in their respective roles in primary care.

**Table 1.** Characteristics of participating CQLs

**Health board and CQL interview number**	**Sex**	**Years qualified as a GP**	**Years in current practice**	**Years as a CQL**
**Health board one, urban high deprivation**				
1	Female	30	25	2.0
7	Male	31	31	3.5
6	Female	29	22	0.75
4	Female	25	25	6.0

**Health board two, urban affluent/mixed**				
12	Male	17	15	6.0
8	Female	20	13	5.0
5	Female	16	6	1.5
2	Male	17	12	3.5

**Health board three, remote and rural**				
3	Female	23	18	4.5
9	Female	13	12	6.0
10	Female	16	4	0.5
11	Female	18	7	2.5

*CQL = cluster quality lead.*

Four specific themes were identified from the interviews:

- support for the aims of the new GP contract and primary care reforms;
- slow progress on expansion of the MDT;
- slow progress on cluster working; and
- lack of focus on older people or on reducing health inequalities.

**Figure 1.**
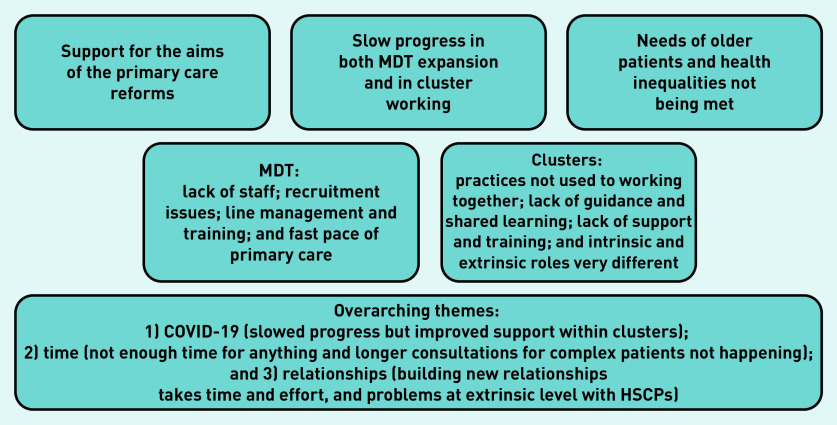
*Findings from thematic analysis of interviews with senior stakeholders and CQLs.* *CQL = cluster quality lead. HSCP = Health and Social Care Partnership. MDT = multidisciplinary team.*

Within these four themes, several subthemes emerged. There were three additional overarching themes — relationships, time, and the effect of the COVID-19 pandemic. The key findings are summarised in Figure 1.

### Theme 1: support for the aims of the new GP contract and primary care reforms

There was general support for the original aims of the primary care reforms, and the majority of PCSs and CQLs believed that ending the QOF was a positive step forward as it was seen as too numbers driven, a tick box exercise, and was too focused on single diseases. The reforms were seen as an opportunity to deliver more person-centred, holistic care:

*‘I think at its inception they* [the reforms] *were a very welcome change. A move away from the QOF mentality of top–down target setting. I thought the CQL movement in terms of quality improvement based on local needs and the grassroots priorities, was very welcome. So a bold, novel approach.’*

(CQL_02, urban affluent/mixed area)

Expanding the MDT workforce was, in principle, welcomed as a means of improving patient care and reducing GP workload. The introduction of GP clusters was seen positively by most:

*‘Widening out the MDT, freeing up more time for GPs to spend with complex multimorbidity patients, delegating other tasks to MDT staff is a good model … replacing QOF with GP clusters, driving quality according to the priorities of their local populations, flexibility of practices being able to develop their MDTs according to the needs of their local population — all a really good idea.’*

(PCS_02)

However, CQLs from remote and rural areas felt that the contract and wider primary care reforms were too ‘city centric’ and did not take into account the unique and diverse challenges of providing primary care services in their areas:

*‘They’re only going to make health inequalities worse because everything is getting centralised — patients are going to get less basic and less personalised services … with the investigation and treatment rooms why should somebody have to do a 50-mile round trip to have three stitches out when there is a GP surgery just around the corner? Not everyone has a car … The cost of travel for our patients, that’s a huge thing.’*

(CQL_03, remote and rural area)

### Theme 2: slow progress on expansion of the MDT

Although there was widespread support for the expansion of the primary care MDT, most interviewees reported that this had not developed at the pace and scale required. As one PCS stated:

*‘I would say there has been some change in MDT working, but it’s certainly not worked on the scale that it was intended to work at. The reality is that the workforce is not there to extend the multidisciplinary team. There aren’t the pharmacists, physios, or ANPs* [advanced nurse practitioners] *. It’s been a very piecemeal introduction. It’s very patchy across the country.’*

(PCS_01)

Because of this slow progress, almost all interviewees stated that GPs were not able to provide longer consultations for patients with complex needs (one of the key aims of the reforms):

*‘I haven’t had any time released for me to devote to those complex multimorbid patients … For me to be able to do that I have to be able to devote sufficient amounts of time. The reforms have not allowed me, or indeed anybody else as far as I know, to do that.’*

(CQL_07, urban high deprivation area)

However, a number of interviewees gave examples of benefits to patients and practice staff of greater MDT working:

*‘We’ve established a very good APP* [advanced physiotherapy practitioner] *team across all the practices in our cluster. So reduction in onward referral and in analgesics, with high patient satisfaction, high doctor satisfaction. A generally wonderful service. Similarly, primary care mental health nurses have also been wonderful, right person, right place, and right time. It’s all there.’*

(CQL_02, urban affluent/mixed area)

In remote and rural areas, there were additional concerns relating to allocation of staff by practice size:

*‘Expanding the MDT workforce sounded great. Then you discovered they’ll be allocated according to practice population size … Meaning you’ll be eligible for two hours of this (MDT staff) a week. That won’t help at all. The reforms and models just don’t fit rural areas … what we really need is more doctors. If you can’t have MDT staff every day in the practice, the only person that could fulfil all those roles is the GP.’*

(CQL_09, remote and rural area)

Two subthemes on the MDT theme were identified, as shown below (quotes for these subthemes are shown in Supplementary Appendix S1).

#### Subtheme 2a: line managing and training new MDT staff

CQLs in all three sites highlighted disadvantages of new MDT staff being employed and line managed by the HSCPs rather than the actual GP practices they work in. Many also spoke about the significant training and supervision requirements GPs had to provide to new MDT staff, which they felt the new GP contract did not sufficiently address.

#### Subtheme 2b: new MDT staff adapting to the demands of primary care

Interviewees spoke of the challenges that new MDT staff can face when coming into the general practice environment, especially around adapting to the fast paced and constant everyday high demands of working in a GP practice.

### Theme 3: slow progress on cluster working

All interviewees reported poor progress around cluster working. Although COVID-19 clearly had a major impact on the progress of the new contract (see overarching themes below), interviewees reported that progress of cluster working had been slow before the pandemic. Although good examples of cluster working were reported, most felt that clusters had not reached the expected levels of performance and proficiency:

*‘There is a large degree of variance. I think the GP clusters understand why they were set up and more than half have bought in to that. It’s been a few years since set-up. I would say we’re still in the toddler stage. We’re not anywhere near walking.’*

(PCS_06)

*‘So to be totally open on clusters, it’s very slow progress … We’re struggling I think … Getting to the extrinsic stuff — not happening to the degree that we were hoping for. Again it comes back to the time thing.’*

(PCS_03)

This CQL, when asked if they were reaching their potential in regards to fulfilling the CQL role, said:

*‘Not even slightly … I can’t believe that it’s five or six years that we’ve been doing clusters. We’ve never really got major things done when you look at what clusters were set up to do. I don’t feel that we’re anywhere near that … There’s just not enough hours in the day.’*

(CQL_04, urban high deprivation area)

The reasons for this slow progress are further explored in the subthemes below (quotes for these subthemes are shown in Supplementary Appendix S1).

#### Subtheme 3a: a history of limited collaboration between practices

A number of interviewees reflected that, historically, there had often been limited collaboration between GP practices in the same location, and that actually getting GPs together and cooperating in a collegiate manner was one distinct positive outcome of clusters.

#### Subtheme 3b: greater guidance and shared learning is required

Although interviewees supported the Scottish Government’s initial decentralised approach of allowing clusters to develop organically, most felt that after a few years there was not enough guidance from the Scottish Government on the sort of quality improvement activities they should focus on (now that the QOF had been removed), and that most clusters were working in isolation without shared learning. Many interviewees felt that a significant re-assessment and re-booting is now required as primary care emerges out of the pandemic in Scotland.

#### Subtheme 3c: lack of infrastructure support for clusters and training for CQLs

Most interviewees felt that the infrastructure and training support for clusters and CQLs were inadequate. This included a lack of useful primary care data, health intelligence, support with analysis, training in quality improvement, leadership training, and evaluation. Interviewees also highlighted the variation in the protected time that CQLs have.

#### Subtheme 3d: challenges in fulfilling the intrinsic and extrinsic role of clusters

Interviewees reported more progress on the intrinsic role (quality improvement within the practices in the cluster) than the extrinsic role (contributing to the planning and development of integrated care in the HSCP/IJB). CQLs with previous leadership roles felt more able to contribute to the extrinsic function than new CQLs without such experience who felt they needed training and support for this role. Several interviewees also commented on the apparent reluctance of the HSCPs and the IJBs to engage with the CQLs. Many CQLs and PCSs noted that the intrinsic and extrinsic roles of clusters required quite different skill sets (and perhaps different personalities).

### Theme 4: lack of focus on older people or on reducing health inequalities

In terms of meeting the needs of older patients or those experiencing health inequalities, the overwhelming view was that progress to date was very limited or absent:

*‘What’s remarkable is even with our new workforce we still haven’t found the capacity for 15-minute appointments, and that’s what we really need for those more complex patients. We’ve never achieved that because we’re too busy … Our workload has not gone down.’*

(PCS_01)

*‘I think there’s a recognition of the growing gap with regards to health inequalities. COVID has really put that under the spotlight. I think we’re still asking the questions. I don’t think we’re beginning to come up with the answers yet.’*

(PCS_04)

The lack of progress on health inequalities was especially emphasised by CQLs in the clusters in deprived areas:

*‘No, it hasn’t released any more time for me to devote to those complex multimorbid patients. For me to be able to do that I have to be able to devote sufficient amounts of time, which myself and others don’t have.’*

(CQL_07, urban high deprivation area)

*‘The GP reforms — they are not there to support people who are in deprived areas. I don’t think deprivation has been considered at all … The deprived patients are not getting any more help, any more support, any more anything. In fact they are getting less because of COVID.’*

(CQL_06, urban high deprivation area)

*‘The additional resource through the SAF* [Scottish Allocation Formula] *went more to affluent practices than more deprived practices, because the allocation of formula was weighted towards age. So if you’ve got older populations, you generally have more affluent populations. So the new contract has not improved health inequality.’*

(PCS_02)

### Overarching themes

#### Relationships

Interviewees spoke frequently of the crucial importance of good relationships across a range of fronts. CQLs saw relationship building as a two-way process and many believed that the IJBs and HSCPs should be more proactive:

*‘We’ve got a chief executive of the HSCP who I’ve never ever met, and I can’t believe as a CQL that I’ve never met that person. I think those sort of people need to meet us. They need to understand what’s going on with us.’*

(CQL_3, remote and rural area)

*‘It’s easy to create new systems, but those systems will not work well if you don’t also invest in the relationships that underpin those systems.’*

(PCS_02)

*‘It does require quite significant teamwork. It means collaborative working across the whole interface. So that’s the relationships with clusters and your local HSCP … the HSCPs and the cluster leads — they need sound relationships, also the relationships with your PQLs. Building relationships takes time and it is a very iterative process.’*

(CQL_02, urban affluent/mixed area)

Pre-existing relationships were beneficial in this wider, extrinsic role:

*‘A lot of the work that’s gone well has been built on existing relationships … Where there’s already rooted relationships across health, social care and primary care it’s been smoother. Where relationships hadn’t been previously cemented, it’s been a little bit more challenging.’*

(PCS_06)

Another key area of relationships was between GP practices and patients. Good patient relationships were regarded as central to good general practice, and there were concerns that some of the changes, such as patients being triaged by reception staff to a new MDT member, might damage these relationships. The majority of interviewees also felt that communication with the public about the primary care reforms had been inadequate:

*‘You’ve got to invest in taking the public with you if you’re going to make big changes in primary care. Otherwise they feel that things are being taken away from them. There is a real need for ongoing reassurance and communication. A lot of work still needs to be done around helping people to navigate these new models of care.’*

(PCS_02)

#### Time

Lack of time was regarded as a major barrier to delivering on some of the key aims of the contract, notably on GPs working as expert medical generalists in extended consultations for complex patients, and CQLs having the time to fulfil the intrinsic and extrinsic roles of clusters. For many interviewees, the shortfall in GP numbers and insufficient numbers of MDT staff were significant factors driving these time pressures:

*‘If you want the cluster leads to do more we still need other GPs to do their work and free up their time. The external role of CQLs has barely started. I just don’t think they’ve got enough time. I think a lot of clusters have struggled with having enough time.’*

(PCS_01)

Time pressures were frequently cited by CQLs as negatively impacting on their ability to fulfil the basic CQL role, as these CQLs noted:

*‘Clusters have been around for many years and we’ve not produced enough. But then I don’t know that we’ve got the time to do it … At the moment, nothing that has been brought in to try and help to relieve GPs’ time, has actually relieved GPs’ time.’*

(CQL_06, urban high deprivation area)

*‘Most GPs haven’t wanted to be a cluster lead because although there’s a payment for it, it’s all about time. Most GPs have got themselves sucked in to the day-to-day practice demands. A lot of them find it difficult to get above that.’*

(CQL_01, urban high deprivation area)

#### COVID-19

All interviewees reported that the COVID-19 pandemic had had a negative impact on the implementation of the primary care reforms. Although clusters continued to meet (virtually) during the pandemic, the intrinsic and extrinsic functions largely came to a halt as general practice went into ‘survival mode’ and GP clusters then took on a supportive role to help GPs cope. Cluster meetings also usefully facilitated shared learning in coping with the challenges of providing GP services during the pandemic:

*‘If we’re looking at the purposes of the clusters — quality improvement — in the last eighteen months it’s an aspirational thing. We’ve had no time. We’re struggling to do the basic core GP work. Clusters have just been fire-fighting to cope with COVID.’*

(CQL_03, remote and rural area)

*‘Pre-COVID it was a slow march. COVID I would say has halted it. In fact, we’ve probably gone back the way. We’ve lost staff, the remote working has meant we’re not working as a unit anymore. COVID has had a major negative impact on the implementation of the reforms.’*

(CQL_04, urban high deprivation area)

*‘MDT working — we’ve not been able to really do that throughout the pandemic, apart from this brief huddle on a daily basis. In terms of learning together, building teams together and creating strategies together, all of that has had to go on the backburner during COVID.’*

(PCS_02)

*‘I’ve been a GP now for nearly eighteen years and it’s the first time I’ve really talked to other GPs, not in an educational setting or something. Clusters have been positive in that it’s brought the practices together and it’s been very good during COVID supporting each other. That was a huge advantage. Prior to clusters there was a lot of miscommunication, or no communication between practices.’*

(CQL_11, remote and rural area)

A ubiquitous concern was the high volume of work that primary care is facing as Scotland emerges out of the pandemic, and that tackling this backlog of patient care will further hamper attempts to progress the primary care reforms.

### Key learning

Interviewees were asked to reflect on the key learning from the introduction of the GP contract and wider primary care reforms to date. Four key issues emerged. These were, first, the need for a re-assessment of the new Scottish GP contract to promote a faster pace of change in primary care transformation:

*‘My learning is that what they put on paper, we need to start seeing it actually happen. That’s the frustrating part at the moment. There’s a lot of talk and no action.’*

(CQL_04, urban high deprivation area)

*‘I don’t think enough has changed. Which they’ll call negative. I don’t think we are going forward well at all.’*

(CQL_06, urban high deprivation area)

*‘They should be reviewing — saying the ones* [parts of contract/reforms] *that are shown to be of most benefit are the ones that we should be putting the money into. The ones that aren’t of any benefit, the money shouldn’t be going into that.’*

(CQL_06, urban high deprivation area)

Second, there was improved public/patient engagement about the contract/reforms:

*‘Key learning would be you’ve got to invest in taking the public with you if you’re going to make big changes … To be honest there’s still a lot of work that needs to be done around helping people to navigate these new models of care. I think a lot of people still struggle to understand why things have changed.’*

(PCS_02)

*‘How do we get the messaging out to the wider community — that actually you don’t need to see a doctor for x, y, and z.’*

(CQL_05, urban affluent/mixed area)

Third, there was better workforce planning:

*‘If there isn’t the workforce to deliver the strategy, then it can create real difficulties in maintaining trust in the process and maintaining morale, because people feel they’ve been promised something which is then not deliverable.’*

(PCS_02)

*‘Key learning — workforce planning, and accountability for that. Support for training the workforce in the clinical setting. More GPs to allow more time for MDT working, or for review of complex patients, and for cluster working. We need more resource, and more people.’*

(PCS_01)

Fourth, there were better primary care data:

*‘Collect data from the outset so that we know if any of this has made an improvement … we don’t have the data that says, actually, this has had an impact on that population group. If you’re introducing reforms you need to be capturing the data and consistently reviewing that data. For me it’s just been a huge gap in all of this.’*

(PCS_05)

*‘We definitely need more data. I think as a CQL what you would want to do is you would want to have data on the practices. You would want to have specific practice data that you can all look at as a cluster. I need to know what it is like on a practice basis so that we can help identify areas that we need to work on.’*

(CQL_12, urban affluent/mixed area)

## DISCUSSION

### Summary

This qualitative study with key national PCSs and CQLs reveals that, although most supported the aims of the new Scottish GP contract, the majority view is that progress has been slow, and this is only partly because of the pandemic. None of the CQLs (and few PCSs) felt that GP workload had reduced significantly, nor that the care of patients with complex needs had improved. Lack of time and poorly developed relationships were key barriers, as was a lack of adequate support across a range of areas including communication between clusters, guidance on areas of clinical care to focus on, primary care data, health intelligence, analysis, training in quality improvement, leadership training, and evaluation. There was also concern expressed about the large backlog of unmet need post-pandemic, especially by CQLs working in deprived areas.

### Strengths and limitations

A strength of the current study is that, in addition to gaining the views of senior national PCSs, clusters in three health boards were purposively sampled (in clusters serving urban deprived areas; urban mixed areas; and remote and rural areas) to gain the views of CQLs working in these diverse settings. However, because the first 12 clusters that agreed to participate in the programme of research were recruited there may be a sample bias — those who were keen to take part may have held stronger views, either positive or negative, than CQLs as a whole. The gathering of views through telephone interviews (which was a necessity because of the pandemic) may have been less valuable than face-to-face because of lack of body language and facial cues, for example, although studies have suggested that the telephone is either as good as face-to-face^18^ or in some circumstances actually better.^19^

A further limitation in this study is that only the views of PCSs and CQLs were sought; however, in the next phase of this work the authors will interview other GPs, MDT staff, and patients. Finally, as in all qualitative research, these findings may not be generalisable. However, future work will include a national GP survey in Scotland, which will provide representative quantitative views on primary care transformation, building on the Scottish School of Primary Care’s national GP survey in 2018.^20^

### Comparison with existing literature

To the authors’ knowledge, the current study is the first to include views on MDT working as part of the new GP contract in Scotland and these findings raise questions, not only about the numbers of staff being recruited, but also how effectively new staff are being integrated and embedded into primary care teams and the culture of general practice.

Recent research in England has identified the complexity of matching patients’ needs with practitioners’ capabilities when trying to reduce GP workload by redistributing workload to MDTs.^21^ A recent King’s Fund report on the expansion of MDT staff in primary care networks (PCNs) in England also concluded that *‘while PCNs have swiftly recruited to these roles, they are not being implemented and integrated into primary care teams effectively’*.^22^ Similar barriers to those identified in the current study have also been reported internationally.^23^^,^^24^

The current findings on the continuing slow progress on cluster working is consistent with the authors’ previous research^12^^,^^13^ and with a newly published report by Healthcare Improvement Scotland.^25^ Indeed, concerns raised in a high-level workshop organised by the Scottish School of Primary Care in 2016, that poor infrastructure support would be a barrier to effective cluster working, appear to have been borne out.^26^ The same workshop also predicted that the extrinsic function of the clusters (in which CQLs are expected to contribute and provide local leadership to the planning and development of integrated care within the IJB and HSCP) would be much harder to implement than the intrinsic function (quality improvement in the care provided to the local cluster population of patients).

In England, early evaluation of the 1250 PCNs that were established in July 2019 have also highlighted poor management and infrastructure support.^27^^,^^28^ Indeed, repeated studies of primary care reforms in England note that adequate infrastructure and managerial support are a key determinant of outcomes.^29^^–^^31^ In Wales — where GP clusters were established in 2010 — similar implementation problems were identified,^32^ which resulted in the Welsh Government allocating two tranches of £10 million, with most clusters also being supported by a full-time, senior, non-clinical, project manager (A Lawrie, personal communication, 2022). International experience of clusters echo the needs for adequate support across multiple domains.^33^^,^^34^

### Implications for research and practice

The findings of the present study suggest that faster progress in implementation of the new Scottish GP contract will require increased levels of infrastructure, management, and strategic support for clusters and CQLs. Equally important is public engagement around the changes in primary care and how they have an impact on patient care. Better informed primary care policy decisions and services are unlikely without better collection and use of robust and reliable primary care data, which remains a challenge in Scotland, and better workforce planning is essential to the long-term success of the reforms. Audit Scotland has suggested, *‘NHS and social care workforce planning has never been more important’* .^35^ As other research into redesigning NHS workforces has cautioned, a poorly planned NHS workforce redesign at national level can even result in increased costs and decreased quality of care.^36^

A key consideration in efforts at transforming primary care in Scotland is the balance between bottom–up and top–down approaches, something that has also been highlighted in early evaluation research into the development of PCNs in England.^37^ Although joint guidance was published in June 2019 by the Scottish Government, the Scottish General Practice Committee of the British Medical Association, and the Royal College of General Practitioners, which included issues such as the need for adequate administrative support, it appears that this guidance has not been implemented in any meaningful way.^38^ In their recent review of social care, as Scotland grapples with establishing a National Care Service, the Auditor General highlighted the importance of learning from previous Scottish public sector reform (including health and social care integration), recommending that one of the next steps for the Scottish Government was *‘strong, consistent strategic leadership from the outset’*.^39^ Interestingly, in Alberta, Canada, Leslie *et al* report a gradual move (over 10–15 years) from a local approach to a more centralised ‘accountability’ approach in the evolution of PCNs.^33^ However, the extent to which the Canadian experience is relevant to clusters in Scotland, especially given the Chief Medical Officer’s vision for ‘Era 3 medicine’ and less centralised control,^40^ remains an open question.

Efforts to transform primary care in Scotland through the new GP contract also need to be considered against the backdrop of rising GP workload, which has been documented recently in Scotland^41^ as well as in England.^42^ In Scotland, three-quarters (73.3%) of GPs report that they are struggling to cope with demand and two-thirds (66.8%) report that their current workload is unmanageable, with more than half (57%) reporting worsening workload since the pandemic started.^41^

As also evidenced elsewhere,^43^ many interviewees, especially CQLs in deprived areas, believed the pandemic had exacerbated health inequalities and warned of the large backlog of unmet need awaiting Scottish general practice as it emerges out of the pandemic. Solutions to the inverse care law are now urgently required.^44^ The Scottish Government’s newly published report of a short-life working group on primary care’s potential role in reducing health inequality may lead to new solutions.^45^ Of equal importance, the persistent concerns of remote and rural GPs in Scotland regarding the new GP contract need to be addressed.^46^

In conclusion, senior national PCSs and CQLs in different areas of Scotland report slow progress in implementation of the new Scottish GP contract, for a host of reasons (including but not limited to the pandemic). These findings suggest that there is an urgent need for better workforce planning, more comprehensive data, and more infrastructure support if the new GP contract is to succeed in transforming primary care in Scotland.
